# Drinking Trajectories and Factors in Koreans

**DOI:** 10.3390/ijerph18168890

**Published:** 2021-08-23

**Authors:** Yun-Young Kim, Hyung-Joo Park, Mee-Sook Kim

**Affiliations:** 1 Department of Social Welfare, Jeonbuk National University, Jeonju 54896, Korea; yun2050@jbnu.ac.kr (Y.-Y.K.); p01065027330@gmail.com (H.-J.P.); 2 Division of General Education, Catholic Kkottongnae University, Cheongju 28211, Korea

**Keywords:** alcohol and culture, social drinking, problematic drinking, ecological system theory

## Abstract

This study aimed to explore the drinking culture in Korea by sex, age, household type, occupation, and income level to identify demographic groups with prominent drinking behaviors and factors affecting their drinking. Furthermore, we evaluated recent changes, including those due to COVID-19, in drinking behavior, using data from the Korea Welfare Panel Study from 2010 to 2020. Panel analysis was performed to reveal the effects of material deprivation, depression, and sociodemographic factors on drinking behavior. We used the AUDIT 3 scale including frequency of drinking, average amount of drinking, and frequency of excessive drinking. The two characteristics of Korean drinking are consistent with the claim of the ecological system theory that humans, as social beings, drink to facilitate social communication or promote problematic drinking when social communication is difficult. Drinking among Koreans is characterized by a pattern that alternates between social drinking and problem drinking. Our study recognizes drinking as a social problem that should be managed at social as well as national levels.

## 1. Introduction

Drinking is not only a personal act affecting the individual body, personality, and emotions, but also a social action influenced by various environmental factors, such as family, social culture, and economy, leading to a habit. Harmful alcohol consumption is considered one of the major risk factors threatening the health of the global population [1]. Problematic drinking is, therefore, both a personal problem negatively affecting the physical, mental, and social health of the individual [2,3] and a social problem, causing socioeconomic losses, such as family and interpersonal conflicts, loss of job or social status, loss of access to resources, and social exclusion [4,5]. According to the WHO [5], the AUDIT scale was developed to screen for excessive drinking and in particular to help practitioners identify whether the person has hazardous (or risky) drinking, harmful drinking, or alcohol dependence. In this text, drinking at a level that causes problems in daily life, such as hazardous and harmful patterns of alcohol consumption or binge drinking, is referred to as problematic drinking.

Drinking is largely influenced by social factors, such as social norms and beliefs about the harmful effects of drinking, in each country [6]. According to the Global Health Research Center, tobacco use, high fasting plasma glucose, and alcohol use are the top three factors shortening the healthy lifespan of Koreans [7]. Compared with Japan and China, where alcohol use is the sixth and eighth factor that hinders the lifespan of the general population, respectively, drinking is a relatively serious problem in Korea. Comparing OECD countries, alcohol consumption (annual sales of alcohol per person over the age of 15) from 2016 to 2018 was 8.5, 7.2, and 5.6 L/capita for Korea, Japan, and China, respectively [8], suggesting that alcohol consumption and drinking problems are relatively prevalent in Korea. These are empirical data that show that Koreans’ healthy lifespan is more seriously threatened by inappropriate drinking behavior compared with OECD and Asian comparative countries. Therefore, in this study, the characteristics of the drinking culture in Korea are examined by longitudinal data analysis over ten years, obtaining implications for alleviating the drinking culture.

However, in Korea, drinking is a part of a tolerant, permissive culture, and the harm of drinking tends to be perceived as an individual health risk rather than a social problem. Thus, little effort has been made to understand drinking problems in a social context [9]. Drinking problems must be recognized as a social problem that should be systematically managed at the social and national levels, rather than being limited to individual or family problems.

Jessor [10] reported that an integrated investigation of the relationship between humans and the social environment is necessary to demonstrate that drinking is a product of social communication. In other words, drinking is not caused by a single factor but by the interaction between individual, family, and social factors. Thus, drinking problems need to be analyzed from the multidimensional perspectives of humans in the environment. Moreover, to better understand the drinking patterns of our society, a longitudinal study is necessary to examine changes in drinking behaviors and various factors affecting problematic drinking through panel analysis. This study analyzed the 2010−2020 data of the Korea Welfare Panel, assessing the characteristics of drinking culture in Korea by sex, age, household type, occupation, and income level to identify demographic groups with prominent drinking behaviors and understand the behaviors and factors affecting their drinking. Changes in drinking behavior due to COVID-19 were also evaluated.

## 2. Literature Review

### 2.1. Ecological Theory for Drinking

The ecological theory introduces an ecological perspective to the general system theory and provides a framework for understanding the relationship between humans and the environment. In this theory, humans and the environment interact to form an integrated system by maintaining homeostasis. The theory hypothesizes that the individual as a complete being is understood in social situations and behavior and personality are formed and developed as humans adapt to and modify environmental needs to suit their needs [4,10].

Jessor [10] argued that an integrated investigation of the relationship between humans and the social environment is necessary to demonstrate drinking as a product of human−society communication. Humans are social beings that maintain positive interactions with their environment through social drinking. However, a negative interaction between humans and the environment due to a failure to maintain a goodness-of-fit can hinder personal development and promote drinking, leading to problematic drinking behaviors [11]. As such, the ecological theory provides a crucial conceptual framework for understanding problematic drinking behaviors.

### 2.2. Drinking Behavior of Koreans by Demographic Factors

#### 2.2.1. Drinking Characteristics by Gender

Traditionally, drinking in Korea is part of a strong group culture that tolerates male drinking and is a group behavior that strengthens solidarity. Similar to foreign countries, there are gender differences in drinking motives, and young men often drink socially to promote social relationships, while women predominantly drink to relieve negative emotions [12,13]. In a panel analysis by Kim et al. [14] using data from the Korea Labor Panel, there were significant differences in drinking behaviors and amounts between men and women. In monthly and high-risk drinking rates for adults from the Korea National Health and Nutrition Examination Survey by the Ministry of Health and Welfare, there was a decreasing trend between 2007 and 2019 in men, after a peak in 2010, while women showed an increasing trend, reaching a peak in 2017 [15]. These results suggest that men showed a higher drinking frequency and were more likely to drink 3−4 times a week or to drink every day than women.

#### 2.2.2. Drinking Characteristics by Age

The Korea National Health and Nutrition Examination Survey by the Ministry of Health showed that the drinking rate of Korean adults in 2019 decreased with age [15]. In 2019, those in their 40 s showed the highest asymmetric bell curve for high-risk drinking rates [15]. However, the high-risk drinking rate is highest in adults in their 40 s, while this behavior declines in other age groups.

#### 2.2.3. Drinking Characteristics by Household Type

In recent years, the number of single-person households has increased rapidly in Korea. According to a press release by Statistics Korea, in 2019, three in ten (30.2%) were single-person households in Korea [16]. In multi-person households, drinking behaviors have decreased, due to pressure from family members [15,16]. In contrast, single-person households have less social control over health risk behaviors, leading to increased drinking opportunities [17]. Analysis of annual change trends by Kim et al. [14] (p. 44) showed that single-person households were more likely to drink 3–4 times a week or to drink every day compared with multi-person households in most years between 2007 and 2015. In addition, panel analysis by Kim et al. [14] (p. 47) demonstrated that drinking frequency and drinking 3–4 times a week were significantly greater in single-person households.

Yoon and Lee [18] analyzed the Korea Welfare Panel data and reported that the 1-year prevalence of alcohol use disorder in single-person households was greater than in the general population. Furthermore, the increase in the drinking level of single-person households was associated with an increased level of depression. In previous studies, family factors, such as marital status, family relationships, conflict, and cohesion, significantly affected problematic drinking more than individual factors [11,19,20]. Finally, heavy drinking was more common in those who were not married [19].

#### 2.2.4. Drinking Characteristics by Occupation and Income Level

The drinking culture in foreign countries is such that lower income is associated with increased drinking. According to the World Health Organization (WHO), groups with lower socioeconomic status are more vulnerable to problems caused by drinking [21]. In foreign countries, the proportion of problematic drinkers commonly decreases as social class or socioeconomic status increases, and vice versa [22]. Those with a low income are more likely to either abstain from drinking altogether or show heavy alcohol consumption than they are to drink a moderate amount [23]. An annual income below the median increases the chance of heavy alcohol consumption [19].

The Korea National Health and Nutrition Examination Survey by the Ministry of Health showed the monthly drinking rate for Korean adults in 2019 was highest in those with a mean income level, followed by those with high, high-median, median-low, and low-incomes [15]. Kim et al. [14] conducted panel analysis using data from the Korean Labor Panel, showing that income had significant effects on drinking frequency, suggesting that, in Korea, higher income was associated with greater drinking. However, in the 2019 Korea National Health and Nutrition Examination Survey, the rate of high-risk drinking was highest in those with median-low, low-median, high, and high-median incomes [15]. Kim et al. [14] reported similar findings, noting that daily drinking was the highest and lowest in those whose income was in the first and fifth quintile, respectively, suggesting a positive relationship between daily drinking and ranking of income decile. Altogether, these findings suggest that, in Korea, the monthly drinking rate is higher with increased income, while the high-risk drinking rate and daily drinking rate are low.

#### 2.2.5. Relationship between Drinking and Satisfaction with Life

Khan et al. [24] found that poverty in Canada is positively associated with alcohol use and drinking problems. Unemployment temporarily decreased alcohol use; however, prolonged unemployment led to increased alcohol use. Kim and Kim [25] analyzed 6-year longitudinal data from the Korean Welfare Panel and observed that socioeconomic deprivation, encompassing dietary life, residential environment, occupational relationships, family relationships, social relationships, and leisure life, led to problematic drinking in Korean adults. Furthermore, Lee and Lee [26] (p. 557) analyzed the Korea Welfare Panel data from three different years, reporting that material difficulties of low-income individuals had significant effects on drinking, mediated by family conflict and self-esteem.

#### 2.2.6. Relationship between Depression and Drinking

Mental disorders, including depression, are closely related to excessive alcohol consumption. Simultaneous experiences of a psychiatric disorder and substance abuse are referred to as dual diagnoses or co-occurring disorders [27]. Drinking to cope with depression can lead to alcohol abuse [28]. In a study of patients in psychiatric hospitals, Cheon et al. [29] showed that 74.1% of patients abusing alcohol also had psychiatric disorders, such as anxiety and depression.

#### 2.2.7. Characteristics of Drinking after COVID-19

In 2020, the spread of coronavirus disease 2019 (COVID-19) worldwide brought about changes in people’s daily lives. In some countries, alcohol sales soared as people attempted to cope with stress [30]. The Morning Consult reported that alcohol consumption in 2020 after COVID-19 increased by 25% in millennials, compared with Gen X and Boomers [31]. In another study that assessed the relationship between alcohol use and large-scale crisis events, including COVID-19, the increase in alcohol use was partially mediated by anxiety, depression, and post-traumatic stress disorder (PTSD) [30]. Demographic studies indicated that those who are young and unmarried are more vulnerable to risky drinking behaviors after a crisis.

In the UK, factors related to drinking behavior during social distancing and local lockdown due to COVID-19 were assessed, and drinking in young age groups was found to increase (compared with times before the COVID-19 pandemic) [32]. Similarly, in Korea, the National Mental Health Survey by the Ministry of Health and Welfare and the Korea Society for Traumatic Stress Studies [33] showed that the frequency and amount of alcohol consumption decreased concurrently after the outbreak of COVID-19. These results may be related to prolonged social distancing impacting upon social drinking.

## 3. Method

### 3.1. Database: Korea Welfare Panel Study (KoWePS)

Here, we analyzed data from the Korea Welfare Panel Study (KoWePS) from the 5th year (2010) to the 15th year (2020) (11 years of data). The Korea Welfare Panel Data is a survey that dynamically assesses changes in the size and living conditions of the poor, working poor, and near-poor, in which the household type, income level, and employment status are rapidly changing. Thus, the KoWePS database dynamically identifies the living conditions and welfare needs of various population groups according to age, income level, and economic activity. The data contain accumulated information on the drinking behavior of Koreans and their factor variables over a long period, which would be suitable for this study.

### 3.2. Variables

The WHO uses tools such as the Alcohol Use Disorder Identification Test (AUDIT) and Cut, Annoyed, Guilty, Eye-opener (CAGE) to measure alcohol abuse and problematic drinking. The Korea Welfare Panel also evaluates 14 drinking-related questions that consist of 10 items on AUDIT-K and 4 on CAGE. AUDIT-K assesses frequency of drinking, the average amount of drinking, and frequency of excessive drinking and seven items on negative experiences after drinking. Previous studies conducted in Korea and other countries reported that AUDIT 3 (frequency of drinking, average amount of drinking, and frequency of excessive drinking) is effective in screening problematic drinking compared with the 10 items of AUDIT [34,35,36]. Therefore, as the dependent variable of this study, AUDIT 1(a1), 2(a2), and 3(a3) assessed the average frequency of drinking, the number of glasses per instance of drinking, and frequency of excessive drinking, respectively. The AUDIT 2 and AUDIT 3 scales belong to the problem drinking category.

**Figure 1:**
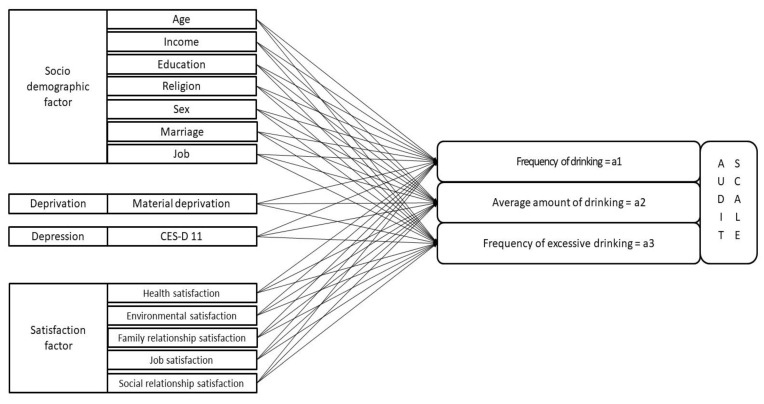
Research Framework.

In addition, independent variables of drinking in Koreans included age, household, sex, income level, education level, religion, marital status, and occupation, based on the literature review in Section 2. The deprivation index was measured using five items on food, housing, credit, medical, and education deprivation, based on the material deprivation questionnaire of the Korea Welfare Panel Survey. It was established by modifying Townsend’s deprivation items [37] to reflect the Korean environment. For the depression score, the Korean Welfare Panel investigated 11 items from the international standard CESD. Thus, this scale was added to calculate the depression scale score with a maximum score of 60 points. This study included studies conducted in 2020 during the COVID-19 spread. The 2020 data survey period was from June 5 to September 24, a period in which Korea was in the midst of the pandemic [38]. Although it is not complete, it is possible to infer the situation of COVID-19 to some extent through the 2020 drinking behavior evaluation. The research model and described variables are shown in Figure 1 and Table 1, respectively.

### 3.3. Panel Analysis

The time-series trends of the main variables related to drinking were investigated, and panel analysis was performed using the STATA 16 package (StataCorp LLC, College Station, TX, USA) to identify the factors of the three variables of the AUDIT scales. Panel data allow us to observe the dynamic trends between variables beyond static relationships at a specific time point [39]. For example, in this study, we observed the time-series trends of various indicators related to drinking (input of analyzed variables). Second, the unobserved heterogeneity factor of individuals can be estimated using this model. To control the unobserved heterogeneity of the described variables, error terms were grouped into variables that differed between individuals (members of the welfare panel’s household in this study) but did not change with time; variables that vary over time but did not differ between individuals; and variables that differ between individuals and change over time [40].

Thus, the random-effects model was used because it satisfied the assumption of uncorrelated heterogeneity by checking the estimated coefficient of time-fixed variables (sex) and controlling for time-fixed individual factors. The estimated panel model was as follows:

(1) $$Y_{i\cdot t}=\alpha +X_{i\cdot t}\beta +\epsilon_{i\cdot t}$$

(2) $$\;\mathrm{If},\;\epsilon_{i\cdot t}=\mu_{i}+\lambda_{t}+\upsilon_{i\cdot t},\;i\left(Individual\right)=1,2.....N,\;t\left(year\right)=1,2.....T)$$

*μi* = unobservable individual effects;

*λt* = unobservable time effect;

*νi·t* = remaining stochastic disturbance term.

## 4. Results

The frequency of drinking was analyzed in a time series from 2010 to 2020, as shown in Table 2. The frequency of drinking ranged from “no drinking” to “four times a week.” After summing up the results of all years, no drinking was the most common habit (59.47%), followed by 2−3 times a month (18.08%), 4 times a week (12.12%), and less than once a month (10.34%). In the analysis of the time series, “no drinking” was the highest proportion (54−63%) in all years. The total sample size of the survey was 140,073, with 12,014 in 2010 and 11,209 in 2020.

**Figure 2:**
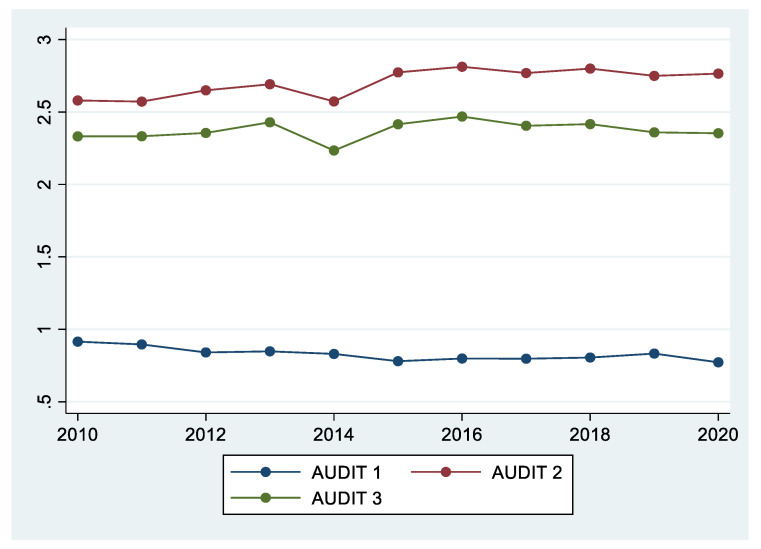
Trends of the three AUDIT items from 2010 to 2020.

Table 3 shows the average values of the three items of AUDIT. For AUDIT 1, a score of 1, 2, or 3 points is equivalent to a drinking frequency of once a week, 2−3 times a week, and 4 or more times a week, respectively. Rather than focusing on the absolute amounts of AUDIT 1, 2, and 3 in this table, it is better to focus on whether each variable has an increasing or decreasing trend over 11 years. In 2010, the average score was 0.91, and in 2020, the average score was 0.77, which was slightly lower than that in 2010. The score for AUDIT 2 and 3 items steadily increased, except in 2014. Overall, the findings suggest that frequency of drinking (AUDIT 1) decreased slightly, while average amount of drinking (AUDIT 2) and frequency of excessive drinking (AUDIT 3) continued to increase until 2015, and it was found that it maintained a certain level from 2016 to 2020. In addition, AUDIT 1 levels were lower in 2020, which means drinking was less frequent due to social distancing. However, unlike AUDIT 1, AUDIT 2 and 3 did not decrease during the same period, and AUDIT 2 average amount of drinking slightly increased. During the COVID-19 pandemic, social drinking with colleagues decreased in terms of frequency, but the average amount of drinking increased once a person started drinking (Table 3, Figure 2).

**Figure 3:**
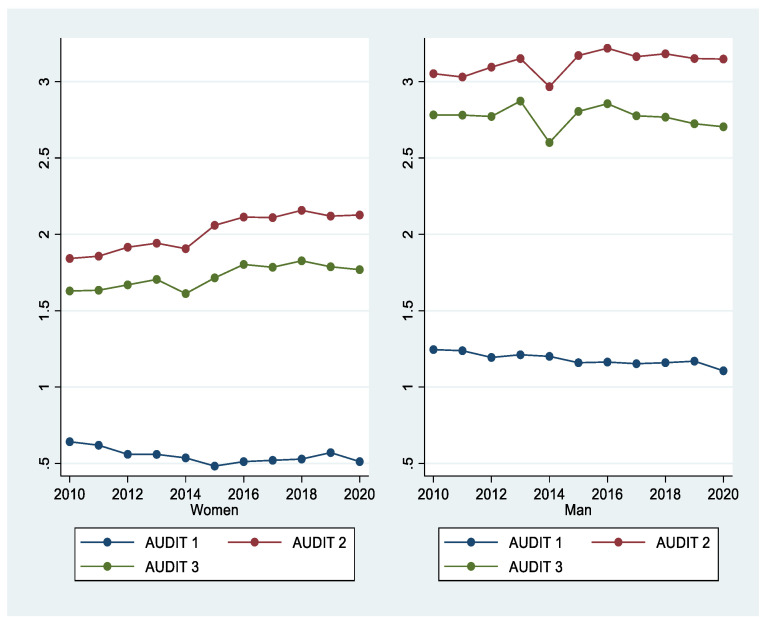
Trends of the AUDIT 1, 2, and 3 items from 2010 to 2020.

**Figure 4:**
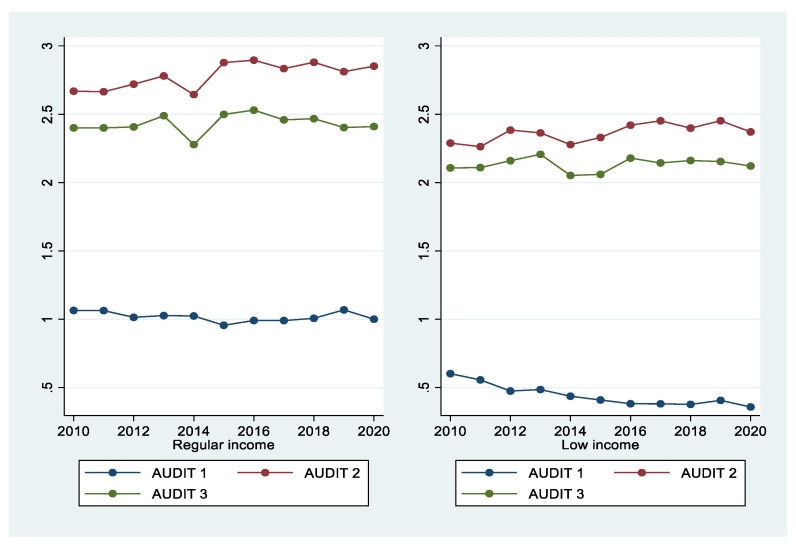
Trends in the three AUDIT items in regular- vs. low-income households from 2010 to 2020.

**Figure 5:**
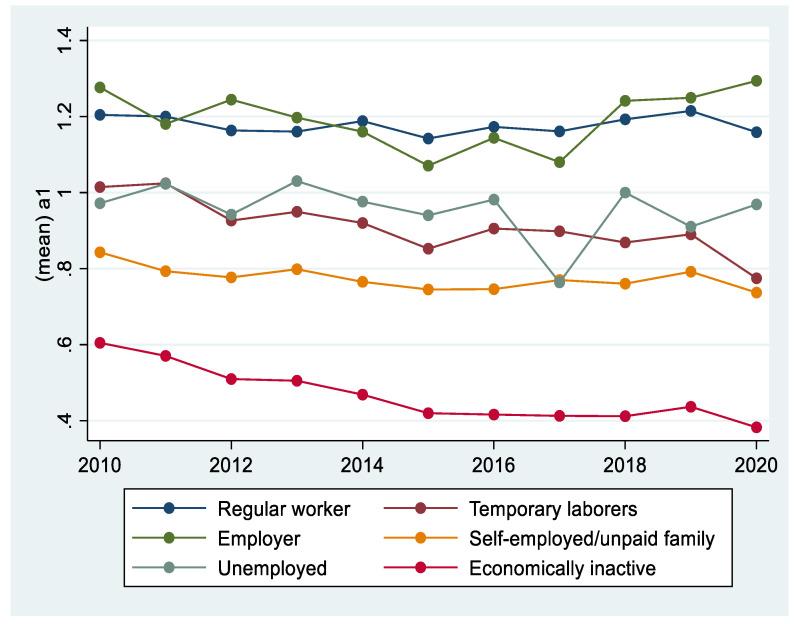
Drinking trends by occupation.

Figure 3 shows the trends of AUDIT 1, 2, and 3 for both men and women. Over the past 11 years, men have generally maintained a higher level of drinking than women. However, AUDIT 3, which indicates hazardous drinking, showed an increasing trend in women. Figure 4 compares and analyzes the three AUDIT items between regular-income households showed a higher level of drinking than low-income households. Figure 5 shows changes in drinking by occupation. Occupations included regular workers, temporary/casual laborers, employers, self-employed/unpaid families, unemployed, and those who are economically inactive. Regular workers and employers, who are relatively more economically stable, demonstrated a higher level of drinking compared with other occupational groups. Drinking was higher in those who were more economically wealthy.

Table 4 shows the results of panel analysis from 2010 to 2020, assessing the effects of demographic characteristics, material deprivation, depression, and psychological and environmental factors on the three AUDIT items (frequency of drinking, average amount of drinking, and frequency of excessive drinking).

First, increased age was associated with reduced frequency of drinking, average amount of drinking, and frequency of excessive drinking. Moreover, higher income and education levels were associated with a higher frequency of drinking. In contrast, education level showed a significant negative relationship with the frequency of excessive drinking and the average number of drinking. Compared with non-religious individuals and women, religious individuals and men showed a higher frequency of excessive drinking. As for marital status, divorced, bereaved, and separated groups drank more than those who were married. In addition, single-person households had a higher frequency of drinking, number of drinks, and frequency of excessive drinking compared with multi-person households. Regular workers and employers showed a tendency for increased frequency of drinking and problematic drinking.

The increased total deprivation index had significant positive effects on the frequency of drinking and frequency of excessive drinking. It showed that low-income households consumed less alcohol, while deprived households were more likely to have problematic drinking behaviors. As depression increased, the frequency of drinking and problematic drinking increased. Lastly, satisfaction with health and social relationships increased the frequency of drinking, average amount of drinking, and frequency of excessive drinking. Furthermore, the frequency of drinking, average amount of drinking, and frequency of excessive drinking decreased as satisfaction with the residential environment, family relationships, and employment increased.

## 5. Discussion

The main results of this study are as follows: In Koreans, higher income and educational levels were associated with increased frequency of drinking, average number of drinking, and decreased frequency of excessive drinking. Groups with heavy alcohol consumption included men (compared with women), divorced/bereaved groups (compared with married individuals), and those with high total deprivation and depression indices. These findings are in agreement with the WHO [1] and the Korea National Health and Nutrition Examination Survey [15]. In addition, the risk of drinking may be higher in underprivileged groups. Thus, more multidimensional health policies would be necessary, rather than focusing on the income quintile alone.

In the 2020 data that reflect the COVID-19 pandemic, the frequency of drinking was reduced; however, the average amount of drinking further increased. This finding suggests that although people drank less often due to social distancing, average amount of drinking consumed on each occasion increased compared with times before the COVID-19 pandemic. Therefore, drinking factors were not unilinear and showed different patterns according to the frequency of drinking, average amount of drinking, and frequency of excessive drinking.

Therefore, this study suggests two different kinds of policy implications to improve the drinking culture in Korea. First, continuous efforts are needed to amend the drinking culture, which encourages social drinking. According to the research results, problem drinking among Koreans is not just a problem of the underprivileged. In addition, policy interest and support are required to reduce problematic drinking for single-person households, socioeconomically deprived groups, and those with difficulties in family relationships. Specific policies need to be created for these groups to detect and treat drinking problems early, through annual health checkups for alcoholism. In particular, institutional arrangements are necessary for the treatment of drinking problems within local communities.

## 6. Conclusions

This study is meaningful because various factors of drinking over 10 years were comprehensively assessed. The characteristics of Korean drinking revealed through the panel analysis in this study (Table 4) are in line with the claim of the ecosystem theory, that human beings, who are social beings, drink alcohol to facilitate social communication and/or fall into problematic drinking when social communication is difficult. Therefore, the following implications were derived from this study. First, in Korea, drinking is a socially acceptable part of socializing, and drinking together is a social activity. Based on the drinking and ecological system theory of Jessor [10], male regular workers and employers showed a tendency for a high frequency of drinking and problematic drinking to maintain social relationships. This finding is contrary to other studies, where lower income was associated with an increased frequency of drinking and problematic drinking [6,21]. In this study, the average number of glasses per instance of drinking (a2) of regular workers was higher than that of other occupational groups. In addition, higher satisfaction with social acquaintances was associated with higher a1, a2, and a3. These findings suggest that drinking is still a way to maintain social relationships in Korea.

Second, single-person households, material deprivation, and dissatisfaction with family relationships were highly related to drinking among Koreans. The a1, a2, and a3 were significantly greater in single-person households, and residential environment satisfaction and family relationships significantly decreased a1, a2, and a3. Moreover, material deprivation and depression were factors that increased the average amount of drinking (a2). These findings were in contrast with previous studies that showed that multi-person households tended to have lower drinking behaviors due to the control and persuasion of family members [41], and the relationship between drinking and depression/drinking and material deprivation tends to be different from that of multi-person households [42].

These two drinking characteristics in Koreans are consistent with the ecological theory, which suggests that humans, as social beings, drink to encourage and facilitate social communication, and difficulties in social communication (e.g., being in a single-person household, social deprivation, problems in family relationships) promote problematic drinking behaviors [11]. This longitudinal study assessing the drinking characteristics of Koreans is significant, as it investigated the two aspects of social drinking (drinking for social communication and problematic drinking of those with difficulties in social communication) explained by ecological theory. However, a negative interaction between humans and the environment due to failure to maintain a goodness-of-fit [43] can hinder personal development and promote drinking, leading to problematic drinking behaviors [11]. As such, the ecological theory provides a crucial conceptual framework for understanding problematic drinking.

Third, drinking trends have changed since the onset of the COVID-19 pandemic. The pandemic has brought about considerable changes in the daily lives of people around the world. Following social distancing, the pattern of drinking has also changed. It was expected that social drinking would decrease, and problematic drinking in groups with poor goodness-of-fit would worsen. Many people hope to return to their pre-pandemic daily life with the help of vaccines; however, as mutations of the virus are being observed, new lifestyles and drinking patterns are expected to continue for the time being. Micallef [44] reported that the 2020 pandemic affected the rapid growth of home alcohol consumption, such as at-home cocktail making, and this alcohol consumption trend is expected to continue. It suggests that the continuation of the pandemic may aggravate problematic drinking in those with difficulties in social communication, rather than drinking for social communication.

## Figures and Tables

**Table 1:** Composition of variables.

Variable	Categories	Explanation
Dependent variables	Three items of AUDIT	
	Average alcohol consumption per year (frequency of drinking)	No drinking = 0, once a week = 1, 2–3 times a week = 2, 3–4 times = 3
	Number of glasses per session of drinking (average amount of drinking)	1–2 glasses = 1, 4–5 glasses = 2, 5–6 glasses = 3, 7–9 glasses = 4, more than 10 = 5, do not know/no answer = 9
	Instances of drinking more than six glasses at a time (frequency of excessive drinking)	Never = 1, once every few months = 2, 1–2 times a month = 3, 1–2 times a week = 4, almost every day = 5, does not know/no answer = 9
Independent variables	Individual Characteristics	
	Age	Less than 35 years old = 1, 35–49 years old = 2, 50–64 years old = 3, more than 65 years old = 4
	Household type	Regular household = 1, low-income class = 2
	Disposable income	Disposable income
	Education level	Preschool (under 7 years old) = 1, no education (older than 7 years old) = 2, elementary school = 3, middle school = 4, high school = 5, professional school = 6, university = 7, graduate school (Master’s) = 8
	Religion	Yes = 1, no = 0
	Sex	Men = 1, women = 2
	Marital status	Not applicable (under 18 years old) = 0, married = 1, bereaved = 2, divorced = 3, separated = 4, single (over the age of 18, single mothers) = 5, others (deaths, etc.) = 6
	Occupation	Full-time = 1, casual labor = 2, employer = 3, self-employed/unpaid family = 4, unemployed = 5, no economic activity = 6
	Deprivation (material deprivation total)	
	Food deprivation	Often = 1, sometimes = 2, not at all = 3, does not know/unspecified = 4
	Housing deprivation	Yes = 1, no = 2, not applicable = 3
	Medical deprivation	Yes = 1, no = 2, not applicable = 3
	Education deprivation	Yes = 1, no = 2, not applicable = 3
	Credit deprivation	Yes = 1, no = 2, not applicable = 3
	Depression	
	Depression level (CES-D) Questions: 1. I did not feel like eating; my appetite was poor; 2. I felt I was just as good as other people; 3. I felt depressed; 4. I had trouble keeping my mind on what I was doing; 5. My sleep was restless; 6. I felt lonely; 7. I was happy; 8. People were unfriendly; 9. I felt sad; 10. I felt that people disliked me; 11. I could not “get going”	Rarely or none of the time(less than 1 day) = 0, some or a little of the time (1–2 days) = 1, occasionally or a moderate amount of time (3–4 days) = 2, most or all of the time (5–7 days) = 3
	Psychological and environmental characteristics	
	Health satisfaction	Very dissatisfied = 1, dissatisfied = 2, neutral = 3, satisfied = 4, very satisfied = 5
	Residential environment satisfaction	
	Family relationship satisfaction	
	Job satisfaction	
	Social acquaintances satisfaction	

**Table 2:** Drinking frequency in 2010−2020.

Year	No Drinking	Once per Week	2–3 Times per Week	4 Times per Week	Total
2010	6526 54.32%	1546 12.87%	2383 19.84%	1559 12.98%	12,014
2011	6320 55.59%	1386 12.19%	2190 19.26%	1472 12.95%	11,368
2012	8659 59.00%	1457 9.93%	2803 19.10%	1757 11.97%	14,676
2013	8241 57.88%	1623 11.40%	2671 18.76%	1702 11.95%	14,237
2014	8048 58.8%	1538 11.24%	2479 18.11%	1622 11.85%	13,687
2015	8343 62.38%	1220 9.12%	2224 16.63%	1588 11.87%	13,375
2016	7880 61.05%	1253 9.71%	2271 17.60%	1503 11.64%	12,907
2017	7671 61.26%	1191 9.51%	2192 17.50%	1469 11.73%	12,523
2018	7489 61.36%	1120 9.18%	2083 17.07%	1513 12.40%	12,205
2019	7049 59.38%	1206 10.16%	2174 18.31%	1443 12.15%	11,872
2020	7074 63.11%	939 8.38%	1871 16.69%	1325 11.82%	11,209
Total/ average	83,300	14,479	25,341	16,953	140,073
	59.47%	10.34%	18.08%	12.12%	

**Table 3:** Trend of the three AUDIT items from 2010 to 2020.

Year	AUDIT 1 (Frequency of Drinking)	AUDIT 2 (Average Amount of Drinking)	AUDIT 3 (Frequency of Excessive Drinking)
2010	0.91	2.58	2.33
2011	0.90	2.57	2.33
2012	0.84	2.65	2.36
2013	0.85	2.69	2.43
2014	0.83	2.57	2.23
2015	0.78	2.77	2.42
2016	0.80	2.81	2.47
2017	0.80	2.77	2.40
2018	0.80	2.80	2.42
2019	0.83	2.75	2.36
2020	0.77	2.76	2.35
Total	0.83	2.70	2.37

**Table 4:** Results of panel analysis.

Variables	AUDIT 1	AUDIT 2	AUDIT 3
Age	−0.0121 ***	−0.0200 ***	−0.0142 ***
	(0.00)	(0.00)	(0.00)
Income	3.09 × 10^−6^ ***	1.67 × 10^−6^ **	1.03 × 10^−6^
	(0.00)	(0.00)	(0.00)
Education	0.110 ***	−0.0327 ***	−0.0555 ***
	(0.01)	(0.01)	(0.01)
No Religion	0.0518 ***	0.0877 ***	0.105 ***
	(0.01)	(0.01)	(0.01)
Male	−0.615 ***	−1.268 ***	−1.114 ***
	(0.01)	(0.02)	(0.02)
Divorce, Bereaved, Separation	0.0611 ***	0.0853 ***	0.0508 **
	(0.01)	(0.02)	(0.02)
Single (not married)	−0.0517 ***	−0.0608 ***	−0.165 ***
	(0.01)	(0.02)	(0.02)
Single-person household	−0.0568 ***	−0.174 ***	−0.146 ***
	(0.01)	(0.02)	(0.02)
Low income household	−0.0500 ***	−0.0693 ***	−0.149 ***
	(0.01)	(0.01)	(0.01)
Day worker	0.00296	−0.0339 **	0.0127
	(0.01)	(0.01)	(0.02)
Employer	0.0324 **	−0.0135	0.0655 **
	(0.02)	(0.02)	(0.03)
Owner-operator	−0.00115	−0.0664 ***	0.00616
	(0.01)	(0.01)	(0.02)
Unemployed person	−0.0177	−0.0542 *	−0.0121
	(0.02)	(0.03)	(0.04)
Economic inactivity	−0.0919 ***	−0.130 ***	−0.0999 ***
	(0.01)	(0.02)	(0.02)
Total deprivation	9.55 ×10^−5^ *	0.000114	0.000328 ***
	(0.00)	(0.00)	(0.00)
Depression	−0.00125 ***	0.00343 ***	0.00846 ***
	(0.00)	(0.00)	(0.00)
Health satisfaction	0.0224 ***	0.0138 ***	0.0185 ***
	(0.00)	(0.00)	(0.01)
Residential environmental satisfaction	−0.0130 ***	−0.0166 ***	−0.0364 ***
	(0.00)	(0.01)	(0.01)
Family relationship satisfaction	−0.0238 ***	−0.0490 ***	−0.0467 ***
	(0.00)	(0.01)	(0.01)
Job satisfaction	−0.00806 ***	0.00412	−0.0181 ***
	(0.00)	(0.00)	(0.01)
Social relationship satisfaction	0.0373 ***	0.0554 ***	0.0606 ***
	(0.00)	(0.01)	(0.01)
Constant	1.653 ***	4.209 ***	3.665 ***
	(0.03)	(0.06)	(0.07)
Observations	171,036	85,567	64,575
Number of pid	21,795	15,852	13,292

AUDIT 1, frequency of drinking; AUDIT 2, average amount of drinking; AUDIT 3, frequency of excessive Drinking; pid, personal ID (serial number granted to individuals). Standard errors are in parentheses. *** *p* < 0.01, ** *p* < 0.05, * *p* < 0.1.

## Data Availability

Data available at https://www.koweps.re.kr:442/ (accessed on 14 April 2021).

## References

[1] WHO (2018). Global Status Report on Alcohol and Health 2018: Executive Summary.

[2] Rehm J., Ashley M.J., Room R., Single E., Bondy S., Ferrence R., Giesbrecht N. (1996). On the emerging paradigm of drinking patterns and their social and health consequences. Addiction.

[3] Sohn A.R., Park J.E. (2008). Comparison of Sexual Behavior between Binge Drinkers and Non-Binge Drinkers among Korean University Students. J. Korean Alcohol Sci..

[4] Graham K., Bernards S., Knibbe R., Kairouz S., Kuntsche S., Wilsnack S.C., Greenfield T.K., Dietze P., Obot I., Gmel G. (2011). Alcohol-related negative consequences among drinkers around the world. J. Addict..

[5] WHO (2001). AUDIT: The Alcohol Use Disorders Identification Test: Guidelines for Use in Primary Care.

[6] Beard E., Brown J., West R., Acton C., Brennan A., Drummond C., Hickman M., Holmes J., Kaner E., Lock K. (2015). Protocol for a national monthly survey of alcohol use in England with 6-month follow-up: ‘The Alcohol Toolkit Study’. BMC Public Health.

[7] Institute for Health Metrics and Evaluation GBD Country profile: South Korea, Japan, China. http://www.healthdata.org.

[8] OECD Alcohol Consumption. https://data.oecd.org/healthrisk/alcohol-consumption.htm.

[9] Eddie D., White W.L., Vilsaint C.L., Berman B.G., Kelly J.F. (2021). Reasons to Be Cheerful: Personal, Civic, and Economic Achievements After Resolving an Alcohol or Drug Problem in the United States Population. Psychol. Addict. Behav..

[10] Jessor R. (2016). The Origins and Development of Problem Behavior Theory: The Collected Works of Richard Jessor.

[11] Grzywacz J.G., Marks N.F. (2000). Family, work, work-family spillover, and problem drinking during midlife. J. Marriage Fam..

[12] Thombs D.L., Beck K.H., Mahoney C.A. (1993). Effects of social context and gender on drinking patterns of young adults. J. Couns. Psychol..

[13] Lim S.Y., Cho H.S., Lee Y.H. (2005). A Case Study about Female Alcoholic’s Alcohol Addictive Process. Korean J. Clin. Psychol..

[14] Kim Y.Y., Moon J.Y., Kim M.S. (2018). A Panel Analysis on the Change Trends of Drinking Factors in South Korea: Data from 2005~2016 in KLPIS. Health Soc. Sci..

[15] Korean Statistics Information Service (2021). Korea National Health and Nutrition Examination Survey.

[16] Statistics Korea (2021). A Press Release for Single-Person Households with 2020 Statistics.

[17] Knibbe R.A., Drop M.J., Muytjens A. (1997). Correlates of stages in the progression from everyday drinking to problem drinking. Soc. Sci. Med..

[18] Yoon M.S., Lee H.J. (2018). The Effect of Residential Satisfaction, Social Relationship, Drinking on the Depression Among One-person Households. J. Humanit. Soc. Sci..

[19] Karlamangla A., Zhou K., Reuben D., Greendale G., Moore A. (2006). Longitudinal trajectories of heavy drinking in adults in the United States of America. Addiction.

[20] Bijttebier P., Goethals E. (2006). Parental drinking as a risk factor for children’s maladjustment: The mediating role of family environment. Psychol. Addict. Behav..

[21] WHO (2014). Global Status Report on Alcohol and Health 2014.

[22] Beard E., Brown J., West R., Kaner E., Meier P., Michie S. (2019). Associations between socio-economic factors and alcohol consumption: A population survey of adults in England. PLoS ONE.

[23] Cerdá M., Johnson-Lawrence V., Galea S. (2011). Lifetime income patterns and alcohol consumption: Investigating the association between long- and short-term income trajectories and drinking. Soc. Sci. Med..

[24] Khan S., Murray R.P., Barnes G.E. (2002). A structural equation model of the effect of poverty and unemployment on alcohol abuse. Addict. Behav..

[25] Kim M.S., Kim Y.Y. (2018). The Impact of Deprivation and Drinking on Suicidal Behaviors: Analysis of KWPS Data from 2012 to 2017. Korean Care Manag. Study.

[26] Lee J.K., Lee R.H. (2016). Material Hardship and Alcohol Use among Low-income Households in South Korea. J. Korea Contents Assoc..

[27] National Alliance on Mental Illness (NAMI) Dual Diagnosis. https://www.nami.org.

[28] Gonzales V.M., Bradizza C.M., Collins R.L. (2009). Drinking to cope as a statistical mediator in the relationship between suicidal ideation and alcohol outcomes among underage college drinkers. Psychol. Addict. Behav..

[29] Cheon E.J., Lee J.Y., Koo B.H., Moon B.Y., Jeong J.Y., Jeong S.H. (2011). The Relationship of Anxiety and Depressive Symptoms According to the Severity of Alcohol Use in Patients with Alcohol Use Disorder. J. Korean Soc. Biol. Ther. Psychiatry.

[30] Concalves P.D., Moura H.F., do Amaral R.A., Castaldelli-Maia J.M., Malbergier A. (2020). Alcohol use and COVID-19: Can we predict the impact of the pandemic on alcohol use based on the previous crises in the 21st century?. A brief review. Front. Psychiatry.

[31] Morning Consult. https://morningconsult.com/2020/04/06/coronavirus-social-distancing-millennials-eating-drinking.

[32] Garnett C., Jackson S., Oldham M., Brown J., Steptoe A., Fancourt D. (2021). Factors associated with drinking behavior during COVID-19 social distancing and lockdown among adults in the UK. Drug Alcohol Depend..

[33] Ministry of Health and Welfare of South Korea (2021). Korea Society for Traumatic Stress Studies 2020.

[34] Seong J.H., Do H.J., Oh S.W., Im Y.R., Choi J.K., Koweon H.J., Cho D.Y., Lee C.H. (2009). Performance of the AUDIT Alcohol Consumption Questions (AUDIT-C) and AUDIT-K Question 3 Alone in Screening for Problem Drinking. Korean Acad. Fam. Med..

[35] Woo S.M., Jang O.J., Choi H.K., Lee Y.R. (2017). Diagnostic Availability and Optimal Cut Off Score of the Korea Version of Alcohol Use Disorder Identification Test (AUDIT-K), Alcohol Consumption Questions (AUDIT-C) and Question 3 Alone(AUDIT3) for Screening of Hazardous Drinking. J. Korean Acad. Addict. Psychiatry.

[36] Gordon A.J. (2001). Three questions can detect hazardous drinkers. J. Fam. Pract..

[37] Townsend P. (1979). Poverty in the United Kingdom: A Survey of Household Resources and Standards of Living.

[38] Korea Welfare Panel Study (2020). The 2020 Korea Welfare Panel Study (KoWePS) A Descriptive Report.

[39] Min I.S., Choi P.S. (2012). STATA Panel Data Analysis.

[40] Huh T.W., Kim Y.Y. (2021). Triangular Trajectory of Sustainable Development: Panel Analysis of the OECD Countries. Int. J. Environ. Res. Public Health.

[41] Swendsen J.D., Merikangas K.R. (2000). The comorbidity of depression and substance use disorders. Clin. Psychol. Rev..

[42] Greenberg E.S., Grunberg L. (1995). Work alienation and problem alcohol behavior. J. Health Soc. Behav..

[43] Green R.R. (2017). Human Behavior Theory and Social Work Practice.

[44] Forbes The Top Ten Trends Shaping the Adult Beverage Market in 2021. https://www.forbes.com/sites/joemicallef/2021/01/13/after-COVID-the-top-ten-trends-shaping-the-adult-beverage-market-in-2021/.

